# Association between the Infant and Child Feeding Index at 8 months and early childhood neurodevelopment

**DOI:** 10.3389/fnut.2025.1727649

**Published:** 2026-01-14

**Authors:** Xiuxiu Li, Jianan Zhang, Xiaowei Dai, Xuemei Liu, Rui Gao, Xuhua Liu, Min Wei, Li Cai

**Affiliations:** 1 Nanshan Maternity & Child Healthcare Hospital of Shenzhen, Shenzhen, China; 2 Tianhe District Center for Disease Control and Prevention of Guangzhou, Guangzhou, China; 3 Shenzhen Cadre and Talent Health Management Center, Shenzhen, China; 4 Sun Yat-sen University, Guangzhou, China

**Keywords:** birth cohort, feeding practice, Food Frequency Questionnaire, ICFI, neurodevelopment

## Abstract

**Objective:**

To evaluate the association between infant feeding practices, measured by the Infant and Child Feeding Index (ICFI) at 8 months, and early childhood neurodevelopment.

**Methods:**

A total of 705 participants were enrolled from the Shenzhen Birth Cohort Study (SZBC), a prospective longitudinal study. Infant feeding practices at 8 months were assessed via a validated dietary recall method combining 24 h and 7 day recall records. ICFI scores were calculated based on seven components (including breastfeeding and complementary feeding) and dichotomized into qualified (>60% of the total score) versus unqualified groups. Neurodevelopment was evaluated across five domains using the Ages and Stages Questionnaire, Third Edition (ASQ-3) at 8, 12, 18, and 24 months. Multivariable Generalized Estimating Equations (GEE) analyzed the association between ICFI status at 8 months and neurodevelopmental delay across early childhood.

**Results:**

At 8 months, 48.5% of infants had qualified ICFI scores. Across the five ASQ-3 domains, the prevalence of developmental delay between 8 and 24 months ranged from 1.0 to 16.8%. After controlling for confounders, infants in the qualified ICFI group exhibited significantly lower odds of delay in the communication domain (OR = 0.51; 95% CI: 0.35–0.75), the problem-solving domain (OR = 0.55; 95% CI: 0.32–0.95), and the personal-social domain (OR = 0.54; 95% CI: 0.37–0.79).

**Conclusion:**

Higher ICFI scores at 8 months, indicating healthier feeding practices, are associated with reduced risk of neurodevelopmental delays through age two. These findings underscore the importance of promoting targeted feeding guidelines to support early childhood development.

## 1 Background

The first years of life represent a critical window for neurocognitive development, with. Early neurodevelopment significantly influencing subsequent IQ (intelligence quotient) and academic achievement (1). It is proved that early identification and intervention for children experiencing neurocognitive developmental delays can substantially improve long-term developmental outcomes (2–4). Among modifiable factors, Nutritional status during infancy emerges as a pivotal determinant of neurodevelopment (5–8). Essential nutrients including protein, long-chain unsaturated fatty acids, iron, zinc, and iodine, and vitamins A, D, B6, B12, and folic acid play particularly critical roles in brain development. Malnutrition resulting from suboptimal breastfeeding and complementary feeding practice during this sensitive period may permanently affect neurodevelopmental foundations (9–12).

While existing research predominantly examines isolated aspects of infant feeding (either breastfeeding or complementary feeding), comprehensive investigations into the relationship between overall feeding quality and early neurocognitive development remain limited, particularly for infants under 24 months of age. A recent cohort study found that higher dietary diversity at 8 months was associated with better neurodevelopment test performance between 24 and 38 months (13). Cai et al. (14) reported modest but significant benefits of breastfeeding on specific memory and language development during the first 2 years of life. Similarly, a Chinese study in rural western regions demonstrated that healthy eating patterns established within the first 2 years were linked to improved cognitive performance as teenagers (15). However, focusing on any single practice in isolation may provide an incomplete understanding, as it fails to capture the synergistic effects of combined feeding practices on neurodevelopment. Therefore, a composite measure that can holistically evaluate the overall quality of infant feeding is needed to better understand its impact on early neurodevelopment.

To address this gap, the Infants and Child Feeding Index (ICFI) offers a comprehensive assessment tool for evaluating overall feeding quality across different age groups up to 24 months (16). This scoring system incorporates multiple dimensions including breastfeeding practices, bottle-feeding, meal frequency and dietary diversity. By integrating these components, the ICFI provides a holistic measure of feeding quality that captures the very synergies which isolated measures miss. While the ICFI has been adapted to Chinese national feeding guidelines (17), few studies have examined its association with early neurocognitive development. Identifying this association could significantly enhance the clinical utility of ICFI by enabling targeted dietary interventions for children at risk of neurodevelopmental delays.

This study aims to investigate the association between ICFI at 8 months and early neurodevelopment assessed repeatedly between 8 and 24 months of age in infants, with the ultimate goal of informing monitoring protocols and facilitating earlier interventions for at-risk populations.

## 2 Materials and methods

### 2.1 Participants

This study was derived from the Shenzhen Birth Cohort Study (SZBCS), a population-based prospective cohort study registered at ClinicalTrials.gov (NCT03830879), with details presented elsewhere (18). Pregnant women were recruited by SZBC staff during their initial antenatal visit at Shenzhen Nanshan Maternity and Child Healthcare Hospital of China. The primary eligibility criteria for SZBCS were: (1) gestation <19 weeks at enrollment; and (2) an intention to deliver at the study hospital and reside in Shenzhen for long-term follow-up. The enrolled participants subsequently engaged in three face-to-face interviews, one in each trimester of their pregnancy. Following childbirth, a series of follow-up interviews were conducted with both the mother and child. These follow-ups included the administration of questionnaires, developmental assessments, and the collection of biological samples for long-term biobanking. The questionnaires comprehensively covered maternal sociodemographics, lifestyle, dietary habits, mental health, and child health and development. The collected biospecimens included maternal peripheral blood, cord blood, placenta tissue, and child feces. The present analysis, however, is based solely on data obtained from the questionnaires and developmental assessments; the biobanked samples are intended for future analyses and were not utilized in this study. The present analysis applied the following additional criteria to the live births from the parent cohort to define the analytical sample: (1) Availability of complete dietary questionnaire data from the 8 month and/or 12 month follow-ups. (2) Completion of at least two Ages and Stages Questionnaire, Third Edition (ASQ-3) assessments before 24 months. (e.g., at 8, 12, 18, or 24 months).

**Figure 1:**
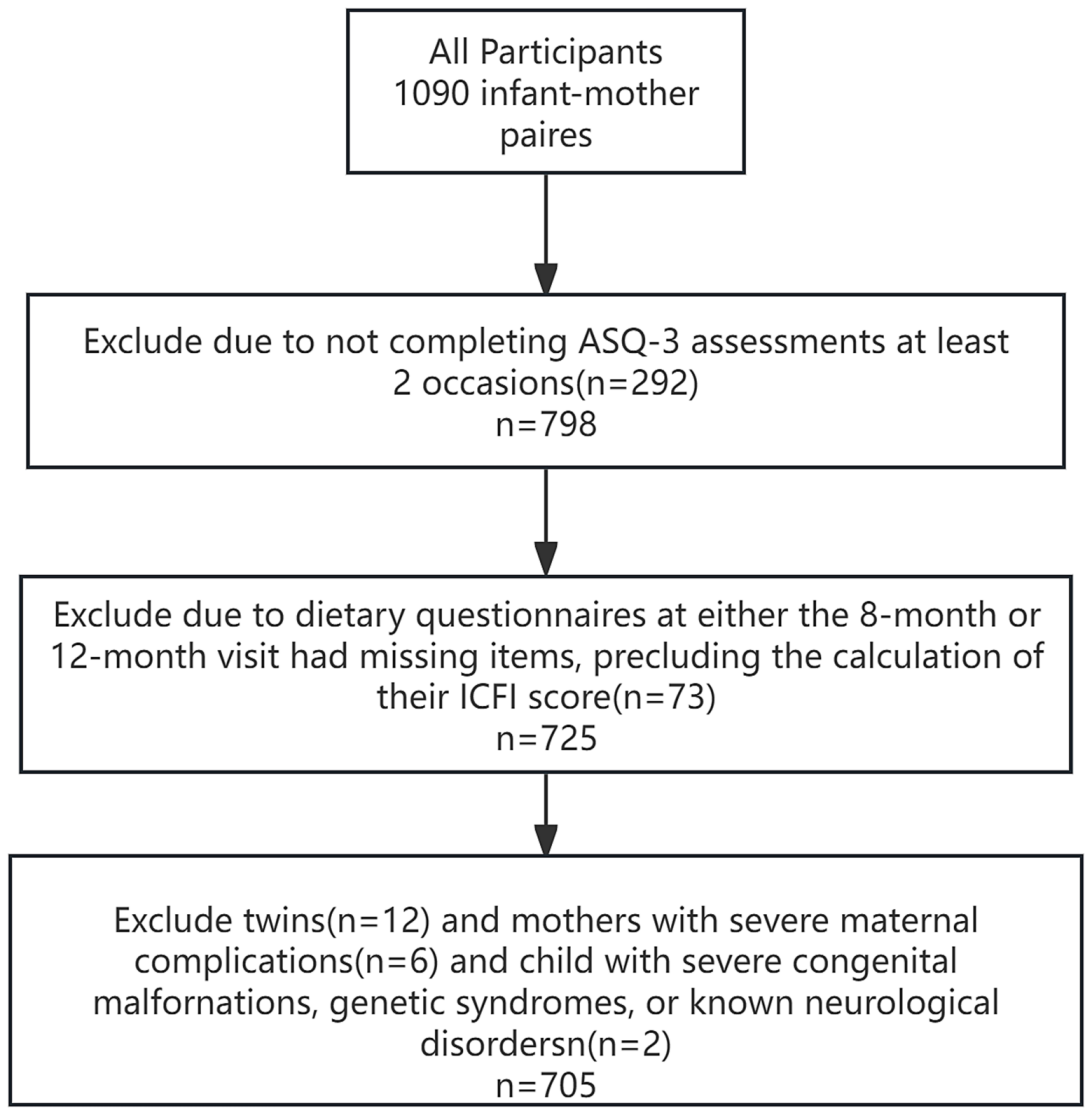
Flow chart showing the number of participants included in the analysis.

Between 2018 and 2021, 1,090 children were included who completed the 24 month follow-up. The derivation of the final analytic sample is outlined in Figure 1. Twins and mothers with severe maternal complications and child with severe congenital malformations, genetic syndromes, or known neurological disorders were excluded. This exclusion was implemented because these conditions are associated with high-risk pregnancies and potential adverse child development outcomes, which could introduce confounding and complicate the interpretation of our primary findings regarding dietary exposures and child growth. Ultimately, our study comprised a sample of 705 children. All mothers provided written informed consent at enrollment. Study protocols were approved by the ethics committees at Nanshan Maternity & Child Healthcare Hospital of Shenzhen (NSFYEC-KY-2020031).

### 2.2 Feeding information and definition

Infant feeding practices were evaluated using a questionnaire administered by trained research personnel, employing a combination of 24 h dietary recall and a validated 7 day Food Frequency Questionnaire (FFQ) (19). The internal consistency of the 7 day food frequency was measured by Cronbach’s *a*, which ranged from 0.68–0.90 across various age intervals. The primary exposure of interest was dietary patterns at 8 months of age, with dietary data at 12 months included in sensitivity analyses to assess temporal consistency. The assessment at 8 months was chosen to capture more stable and diverse dietary patterns compared to the initial transition at 6 months. The validated Infant and Child Feeding Index (ICFI), adapted for the Chinese population, was utilized to quantify feeding quality (20, 21). The initial definition of ICFI was proposed by Ruel and Menon (16), and ICFI scoring system developed by Lai et al. (20) and Yan et al. (21) according to the Nutrition and Health Survey of the Chinese People, and WHO feeding recommendations. This instrument comprises seven core components: (1) current breastfeeding status; (2) bottle-feeding practices; (3) age at initiation of formula supplementation; (4) dietary diversity, defined as the number of complementary food groups consumed within 24 h, which was categorized as grains, vegetables, fruits, proteins (including eggs, poultry, meat, fish, and shrimp), dairy, and legumes, ‘higher diversity’ was defined as consumption of ≥3 food groups; (5) age at introduction of complementary feeding; (6) 24 h feeding frequency of complementary foods; and (7) weekly consumption patterns of specific food categories. The scoring for each of the seven components was performed precisely as outlined in the protocols by Lai et al. (20) and Yan et al. (21), with the full scoring system detailed in Table 1. Each component is scored according to established protocols, yielding a total ICFI score from 0 to 23, where higher scores indicate better adherence to recommended feeding practices. Consistent with Yan et al.’s (21) cutoff, a qualified ICFI is defined as a score >60% of the maximum (i.e., >13.8), whereas an unqualified ICFI is a score ≤60% (≤13.8).

**Table 1:** Components and scores of ICFI by age group.

Variables	Scores	
	6~8 months	12~24 months
Breastfeeding	Yes = 2	Yes = 2
	No = 0	No = 0
Bottle feeding	Yes = 0	Yes = 0
	No = 1	No = 1
Complementary food groups (past 24 h)	1 ~ 2 types = 1	1~2 types = 1
	≥3 types = 2	≥3 types = 2
First complementary feeding age	<4 months or ≥9 months = 0	<4 months or ≥9 months = 0
	4 ~ 5 months = 1	4~5 months = 1
	6 ~ 8 months = 2	6~8 months = 2
Feeding frequency (past 24 h)	No = 0	0–2 times = 0
	1 time = 1	3 times = 1
	≥2 times = 2	≥4 times = 2
Age to start formula milk	<4 months or ≥9 months = 0	<4 months or ≥9 months = 0
	4 ~ 6 months = 1	4~5 months = 1
	6 ~ 8 months = 2	6~8 months = 2
Vegetable	0 ~ 1 time/week = 0	0~3times/week = 0
	2 ~ 3 times/week = 1	4~5 times/week = 1
	≥4 times/week = 2	≥6 times/week = 2
Fruit	0~1 time/week = 0	0~3times/week = 0
	2 ~ 3 times/week = 1	4~5 times/week = 1
	≥4 times/week = 2	≥6 times/week = 2
Egg	0 time/week = 0	0~2times/week = 0
	1 ~ 2 times/week = 1	3~4 times/week = 1
	≥3 times/week = 2	≥5 times/week = 2
Meat	0 time/week = 0	0~2times/week = 0
	1 ~ 2 times/week = 1	3~4 times/week = 1
	≥3 times/week = 2	≥5 times/weeks = 2
Soy	0 time/week = 0	0 time/week = 0
	1 time/week = 1	1 time/week = 1
	≥2 times/week = 2	≥2 times/week = 2
Grain and potato	0~4 times/week = 0	0~5 times/week = 0
	≥5 times/week = 1	≥6 times/week = 1
Milk	0~3 times/week = 0	0~3times/week = 0
	≥4 times/week = 1	4~5 times/week = 1
		≥6 times/weeks = 2
Total scores	23	23
Qualified ICFI	≥13.8	≥13.8
Unqualified ICFI	<13.8	<13.8

### 2.3 Neurodevelopmental assessment

The Ages and Stages Questionnaire, Third Edition (ASQ-3) (22) was administered at 8, 12, 18, and 24 months of age through maternal report. The full questionnaire is provided in the Supplementary material. To account for practical scheduling while maintaining developmental appropriateness, assessments were considered eligible if completed within a predefined window around each target age: ±2 weeks for the 8, 12, and 18 month assessments, and ±4 weeks for the 24 month assessments. This validated parent-completed screening tool demonstrates strong psychometric properties for detecting developmental impairments across five domains: fine motor, gross motor, communication, personal-social functioning, and problem-solving skills. The instrument comprises 30 items (6 per domain), with each item scored as “yes” (10 points), “sometimes” (5 points), or “not yet” (0 points) based on the child’s ability to perform developmentally appropriate tasks. Domain scores were calculated by summing item responses, with higher scores indicating better developmental functioning. A failing score in any domain is a score that is one or more standard deviations below the Chinese standard for that age group, indicating a suspected risk of developmental delay. The ASQ-3 has been widely adopted in pediatric screening contexts, demonstrating moderate-to-high sensitivity (0.70–0.90) and specificity (0.76–0.91) (2, 23). The measure exhibits excellent test-retest reliability (0.94–0.95) and strong inter-rater agreement between parents and professionals (0.94–0.95) (18, 19).

### 2.4 Covariates

During pregnancy, questionnaires were administered to collect data on various characteristics of pregnant women, which were subsequently incorporated in to a multivariable model. The variables included maternal age (<25, 25–29, 30–34, >35), educational attainment (high school technical secondary school or below, college, university, master’s degree or above), monthly family income (<1.5, 1.5–3, 3-4, >4; Unit: RMB 10,000), maternal ethnicity (Han and non-Han), type of household registration (Shenzhen or non-Shenzhen), child’s sex (male or female). Upon birth, the gestational age and birth weight of the newborn were recorded from the birth system. In accordance with the International Newborn Size Standards developed by Oxford University (24), infants with a birth weight below the 10th percentile for their gestational age are classified as Small for Gestational Age (SGA).

### 2.5 Statistical analysis

We compared participant characteristics across ICFI score categories using the chi-square test. Generalized estimation equation (GEE) model (25) was used to explore the association between ICFI (overall scores and component items) and development delays in each of the five ASQ domains (yes/no). This approach accounted for the repeated measures design by incorporating children’s longitudinal ASQ scores at 8, 12, 18, and 24 months.

Odds ratios (OR) and 95% confidence intervals (CI) were estimated for each outcome. The multivariable GEE model adjusted for the following potential confounders: monthly household income, maternal age, education attainment, ethnicity, household registration, parity, delivery mode, child’s sex, and SGA status. These covariates selected based on their known or plausible role as determinants of both infant feeding practices and neurodevelopmental outcomes. In addition to the primary analysis using the dichotomized ICFI variable, a sensitivity analysis was performed by treating the ICFI score as a continuous variable in the GEE models to assess the linear association with neurodevelopmental delays and to verify the robustness of the primary findings. Multicollinearity among the independent variables was assessed for the final model using the Variance Inflation Factor (VIF). All VIF values were below 1.7, indicating no significant multicollinearity.

To address missing covariates data, multiple interpolation (MI), a widely validated statistical technique for handling missing values. Additionally, we conducted sensitivity analyses to evaluate the impact of short-term fluctuations in feeding patterns on developmental outcomes by calculating mean ICFI scores at both 8 and 12 months. This mean score was analyzed both as a dichotomized variable (using the established cutoff of >13.8) and as a continuous variable. All statistical analyses were performed using R (version 4.1.2). All tests were two-sided and statistically significant at *p* < 0.05.

## 3 Results

### 3.1 Population characteristics

The final analytical sample comprised 705 mother-child pairs who had complete data for the infant diet questionnaire at 8 months (enabling ICFI calculation) and at least two completed ASQ-3 assessments between 8 and 24 months of age. Missing data in a small number of covariates were handled using multiple imputation, as described in the methods. The characteristics of the study participants are presented in Table 2. All participating children resided with their mothers at the time of the survey. Notably, Infants who achieved a qualified ICFI score at 8 months, whose mother were more likely with higher education attainment, residing in Shenzhen, and being primiparous.

**Table 2:** Basic characteristics of study participants by infant and child feeding index.

Characteristics	Infant and child feeding index		Chi-square	*p*
	Unqualified (*n* = 342) *n* (%)	Qualified (*n* = 363) *n* (%)		
Maternal age				
<25	19 (5.6)	22 (6.1)	1.3	0.7
25 ~ 29	130 (38.0)	129 (35.5)		
30 ~ 34	133 (38.9)	137 (37.7)		
>35	60 (17.5)	75 (20.7)		
Maternal education				
High schools or below (≤12 years)	29 (8.5)	69 (19.0)	24.0	**<0.001**
College degree (≤16 years)	96 (28.1)	107 (29.5)		
Undergraduate (≤19 years)	166 (48.5)	160 (44.1)		
Master or above (≥20 years)	51 (14.9)	27 (7.4)		
Maternal domicile place				
Shenzhen	237 (69.3)	212 (58.4)	9.0	**0.003**
Others	105 (30.7)	151 (41.6)		
Parity				
Primiparity	216 (63.2)	193 (53.2)	7.2	**0.007**
Multiparity	126 (36.8)	170 (46.8)		
Family monthly earning (CNY)				
<15,000	19 (5.6)	22 (6.1)	1.3	0.7
15,000 ~ 30,000	130 (38.0)	129 (35.5)		
30,000 ~ 40,000	133 (38.9)	137 (37.7)		
>40,000	60 (17.5)	75 (20.7)		
Child gender				
Female	150 (43.9)	159 (43.8)	<0.001	>0.9
Male	192 (56.1)	204 (56.2)		
Delivery mode				
Eutocia	244 (71.3)	238 (65.6)	2.7	0.1
Cesarean	98 (28.7)	125 (34.4)		
Small for gestational age				
No	24 (7.0)	24 (6.6)	0.05	0.8
Yes	318 (93.0)	339 (93.4)		

Bold values indicate statistical significance (*p* < 0.05).

At 8 months of age, 21.3% (*n* = 150) of infants were still breastfeeding (including both exclusive and mixed breastfeeding). However, 57.0% (*n* = 402) of infants had initiated formula feeding by 4 months of age. Among complementary food consumption patterns, 70.8% (*n* = 499) of infants consumed three or more complementary foods within a 24 h period, while over one-fifth (22.1%, *n* = 156) did not receive any complementary foods. Vegetables, fruits, wheat and potatoes were the most frequently consumed food items. Specifically, 50.9% (*n* = 359) and 54.3% (*n* = 383) of children consumed vegetables and fruits on four or more days during the past week, respectively. In contrast, 26.4% (*n* = 186) of infants did not consume eggs, and 37.7% (*n* = 266) did not consume meat (including poultry, red meat, fish, and shrimp) during the same period. Soy products were the least consumed food item, with 74.9% (*n* = 528) of infants reporting no soy consumption in the past week (Table 3).

**Table 3:** Dietary characteristics of 8 month-old infants.

Variable	*N* (%)
Breastfeeding	
Yes	150 (21.3%)
No	555 (78.7%)
Bottle feeding	
Yes	592 (84.0%)
No	113 (16.0%)
Complementary food groups (past 24 h)	
1 ~ 2 types	206 (29.2%)
≥3 types	499 (70.8%)
First complementary feeding age	
<4 months	28 (4.0%)
4 ~ 5 months	177 (25.1%)
≥6 months	500 (70.9%)
Feeding frequency (past 24 h)	
No	156 (22.1%)
1 time	416 (59%)
≥2 times	133 (18.9%)
Age to start formula milk	
<4 months	402 (57.0%)
4 ~ 6 months	232 (32.9%)
6 ~ 8 months	71 (10.1%)
Vegetable	
0 ~ 1 day/week	151 (21.4%)
2 ~ 3 days/week	195 (27.7%)
≥4 days/week	359 (50.9%)
Fruit	
0 ~ 1 day/week	96 (13.6%)
2 ~ 3 days/week	226 (32.1%)
≥4 days/week	383 (54.3%)
Egg	
0 day/week	186 (26.4%)
1 ~ 2 days/week	198 (28.1%)
≥3 days/week	321 (45.5%)
Meat	
0 day/week	266 (37.7%)
1 ~ 2 days/week	178 (25.2%)
≥3 days/week	261 (37%)
Soy	
0 day/week	528 (74.9%)
1 days/week	111 (15.7%)
≥2 days/week	66 (9.4%)
Grain and potato	
0 ~ 4 days/week	443 (62.8%)
≥5 days/week	262 (37.2%)

### 3.2 Distribution of neurodevelopment assessment

**Figure 2:**
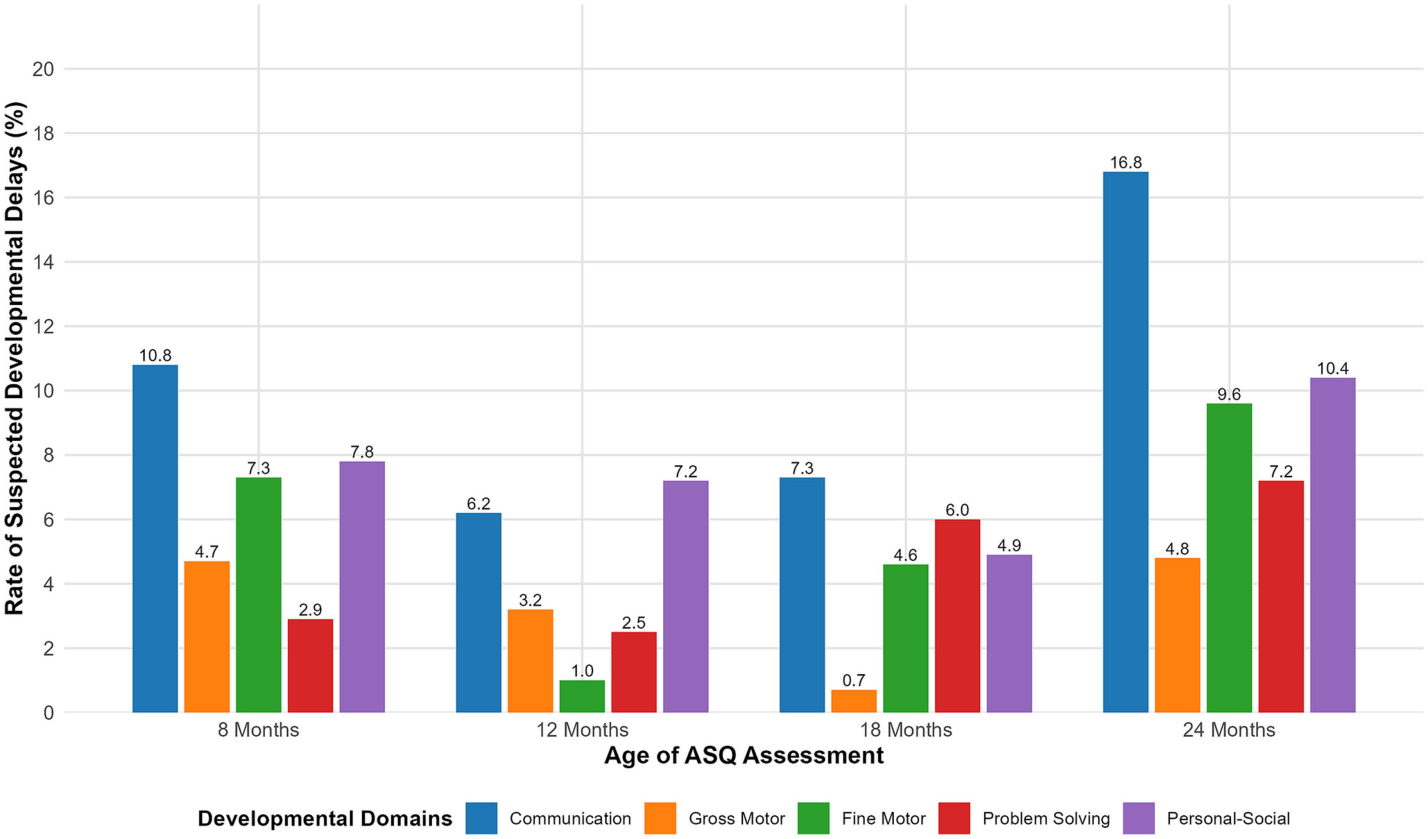
Distribution of suspected developmental delays as measured by the ASQ-3 at different ages. The bar chart depicts the prevalence (as a percentage) of children scoring below the cutoff in any of the five ASQ-3 domains (communication, gross motor, fine motor, problem-solving, and personal-social) at 8, 12, 18, and 24 months of age. Delays are defined as a score falling more than one standard deviation below the Chinese standard mean for each age-specific domain. The total number of children assessed at each time point was 705. ASQ-3, Ages and Stages Questionnaire, Third Edition.

The distribution of completed questionnaires and the prevalence of suspected developmental delays, as assessed by the ASQ-3, across different age groups are presented in Figure 2. Children were most likely to have developmental delays were most frequently observed in communication skills, with 16.8% of children exhibiting delays at 24 months. In contrast, gross motor development demonstrated generally positive outcomes, with only 0.7% of participants showing delays at 18 months of age.

### 3.3 Association between ICFI and infants neurodevelopment

The ICFI score was significantly associated with early neurodevelopment (Table 4). When the ICFI was dichotomized, infants with an ICFI score exceeding 60% of the total score exhibited a reduced risk of developmental delays in communication (OR = 0.51, 95% CI: 0.35–0.75), problem solving (OR = 0.55, 95% CI: 0.32–0.95), and personal-social (OR = 0.54, 95% CI: 0.37–0.79). Analysis of the ICFI as a continuous variable confirmed and refined these findings. After adjustment, higher ICFI scores were significantly associated with a reduced risk of delay in the communication (adjusted OR = 0.93, 95% CI: 0.88–0.98), and personal-social (adjusted OR = 0.91, 95% CI: 0.86–0.96) domains, demonstrating the stability of these associations. Furthermore, this sensitive analysis uncovered a significant protective association with the fine motor domain (adjusted OR = 0.91, 95% CI: 0.85–0.98) that was not detected using the dichotomized variable. For the problem-solving domain, a protective trend was observed which was significant in the crude model but attenuated after adjustment (adjusted OR = 0.93, 95% CI: 0.85–1.02). We further examined the association using the mean ICFI score calculated from both 8 and 12 month assessments to capture sustained feeding quality, the mean ICFI score analyzed similarly showed protective associations across these domains (Supplementary Table 1).

**Table 4:** Association between ICFI scores at 8 months and infants neurodevelopment (*n* = 705).

ASQ	ICFI at 8 ~ 9 months (dichotomized)		ICFI at 8 ~ 9 months (continuous)	
	OR (95% CI)	*p*	OR (95% CI)	*p*
Communication				
Crude	**0.51 (0.36, 0.74)**	**<0.001**	**0.94 (0.89, 0.98)**	**0.017**
Adjusted	**0.51 (0.35, 0.75)** ^a^	**<0.001**	**0.93 (0.88, 0.98)** ^a^	**0.005**
Gross motor				
Crude	0.68 (0.39, 1.18)	0.2	0.94 (0.86, 1.03)	0.208
Adjusted	0.69 (0.40, 1.19)^a^	0.2	0.92 (0.84, 1.01)^a^	0.086
Fine motor				
Crude	0.73 (0.47, 1.15)	0.2	**0.93 (0.86, 0.98)**	**0.021**
Adjusted	0.73 (0.46, 1.15)^a^	0.2	**0.91 (0.85, 0.98)** ^a^	**0.009**
Problem solving				
Crude	**0.53 (0.31, 0.91)**	**0.022**	**0.92 (0.84, 0.99)**	**0.036**
Adjusted	**0.55 (0.32, 0.95)** ^a^	**0.033**	0.93 (0.85, 1.02)^a^	0.114
Personal-social				
Crude	**0.54 (0.37, 0.80)**	**0.002**	**0.92 (0.87, 0.98)**	**0.005**
Adjusted	**0.54 (0.37, 0.79)** ^a^	**0.001**	**0.91 (0.86, 0.96)** ^a^	**0.001**

^a^The ICFI scores <13.8 was used as the control group. Adjusted for monthly household income, maternal age, education attainment, ethnicity, household registration, parity, delivery mode; child’s sex, and Small-for-Gestational-Age (SGA) status. Bold values indicate statistical significance (*p* < 0.05).

To delineate which specific feeding practices contributed to these associations, we further analyzed the individual components of the ICFI (Table 5). In multivariate models, infants who were not bottle-fed had a significantly reduced risk of developmental delays in the communication domain (OR = 0.56, 95% CI: 0.33–0.94) and the problem-solving domain (OR = 0.28, 95% CI: 0.10–0.79) compared to those who were bottle-fed. The addition of formula between 4 and 6 months of age was associated with a decreased likelihood of gross motor developmental delays (OR = 0.44, 95% CI: 0.22–0.88). Higher dietary diversity (consumption of ≥3 food groups) was significantly associated with a reduced risk of delays in fine motor skills (OR = 0.57, 95% CI: 0.36–0.90), problem solving (OR = 0.54, 95% CI: 0.31–0.94) and personal-social development (OR = 0.62, 95% CI: 0.41–0.94). Furthermore, an adequate frequency of complementary feeding (≥2 times/day) was associated with a lower risk of delays in personal-social development (OR = 0.62, 95% CI: 0.41–0.94).

**Table 5:** Association between infant feeding practice and children’s neurodevelopment (*n* = 705).

Variable	Communication	Gross motor	Fine motor	Problem solving	Personal-social
	OR (95% CI)^a^	OR (95% CI)^a^	OR (95% CI)^a^	OR (95% CI)^a^	OR (95% CI)^a^
Breastfeeding (ref: no)	0.70 (0.48, 1.03)	1.24 (0.59, 2.60)	0.83 (0.48, 1.43)	0.74 (0.42, 1.29)	1.18 (0.73, 1.89)
Bottle feeding (ref: yes)	**0.56 (0.33, 0.94)**	0.50 (0.19, 1.32)	0.44 (0.18, 1.09)	**0.28 (0.10, 0.79)**	0.66 (0.37, 1.17)
Complementary food groups (past 24 h) (ref: ≤2 times)	0.69 (0.46, 1.02)	0.92 (0.49, 1.74)	**0.57 (0.36, 0.90)**	**0.54 (0.31, 0.94)**	**0.62 (0.41, 0.94)**
First complementary feeding age (ref: ≤4 months)					
4 ~ 5 months	0.80 (0.33, 1.96)	0.64 (0.13, 3.19)	1.15 (0.34, 3.91)	0.89 (0.24, 3.35)	0.69 (0.24, 1.97)
≥6 months	0.80 (0.35, 1.84)	1.09 (0.24, 4.95)	1.33 (0.43, 4.17)	1.38 (0.40, 4.68)	1.11 (0.42, 2.94)
Feeding frequency (past 24 h) (ref = 0 time)					
1 time	0.75 (0.49, 1.16)	0.74 (0.38, 1.44)	0.73 (0.42, 1.27)	0.74 (0.40, 1.36)	0.65 (0.42, 1.02)
≥2 times	0.70 (0.40, 1.24)	0.58 (0.25, 1.36)	0.64 (0.32, 1.26)	0.61 (0.26, 1.43)	**0.54 (0.29, 0.99)**
Age to start formula milk (ref: ≤4 months)					
4 ~ 6 months	0.97 (0.66, 1.43)	**0.44 (0.22, 0.88)**	0.92 (0.56, 1.50)	1.19 (0.68, 2.09)	0.94 (0.62, 1.42)
6 ~ 8 months	1.03 (0.53, 2.00)	0.85 (0.39, 1.84)	0.87 (0.39, 1.97)	0.98 (0.35, 2.76)	0.89 (0.43, 1.84)
Vegetables (ref: 0~1 day/week)					
2 ~ 3 days/week	1.18 (0.70, 1.99)	0.82 (0.37, 1.81)	**0.41 (0.20, 0.86)**	0.58 (0.28, 1.21)	0.66 (0.37, 1.17)
≥4 days/week	0.84 (0.53, 1.33)	0.77 (0.40, 1.50)	0.77 (0.46, 1.30)	**0.54 (0.29, 1.00)**	**0.60 (0.37, 0.97)**
Fruits (ref: 0~1 day/week)					
2 ~ 3 days/week	0.80 (0.47, 1.34)	**0.29 (0.12, 0.71)**	0.77 (0.39, 1.51)	0.77 (0.38, 1.55)	**0.48 (0.28, 0.81)**
≥4 days/week	**0.54 (0.33, 0.88)**	0.55 (0.29, 1.05)	0.66 (0.35, 1.23)	**0.40 (0.19, 0.81)**	**0.28 (0.17, 0.47)**
Egg (ref: 0 day/week)					
1 ~ 2 days/week	1.02 (0.64, 1.62)	0.56 (0.25, 1.26)	**0.44 (0.24, 0.81)**	1.09 (0.54, 2.20)	0.68 (0.41, 1.13)
≥3 days/week	0.76 (0.49, 1.17)	0.58 (0.30, 1.10)	**0.58 (0.36, 0.96)**	0.96 (0.47, 1.94)	**0.63 (0.40, 0.98)**
Meat (ref: 0 day/week)					
1 ~ 2 days/week	1.19 (0.76, 1.87)	**0.43 (0.20, 0.93)**	0.56 (0.30, 1.03)	0.59 (0.30, 1.17)	0.79 (0.47, 1.32)
≥3 days/week	0.86 (0.56, 1.33)	0.69 (0.36, 1.31)	0.70 (0.42, 1.17)	0.81 (0.45, 1.43)	0.86 (0.55, 1.36)
Soy (ref: 0 day/week)					
1 day/week	0.64 (0.36, 1.13)	0.74 (0.31, 1.78)	**0.47 (0.22, 0.97)**	**0.19 (0.04, 0.77)**	**0.45 (0.23, 0.89)**
≥2 days/week	1.16 (0.66, 2.05)	0.58 (0.21, 1.62)	0.56 (0.23, 1.40)	**2.07 (1.07, 3.99)**	1.05 (0.53, 2.11)
Grain and potato (ref: 0~4 days/week)	0.98 (0.67, 1.44)	0.96 (0.54, 1.71)	1.04 (0.64, 1.69)	1.39 (0.80, 2.41)	0.93 (0.61, 1.42)

^a^Monthly household income, maternal age, education attainment, ethnicity, household registration, parity; delivery mode, child’s gender and SGA were included as covariates in the adjusted models. Bold values indicate statistical significance (*p* < 0.05).

More frequent vegetable intake was associated with a reduced risk of developmental delays in fine motor skills (OR = 0.41, 95% CI: 0.20–0.86), and personal-social development (OR = 0.60, 95% CI: 0.37–0.97) compared to no vegetable consumption during the past week. Similarly, increased fruit consumption was linked to lower risk of developmental delays in communication (OR = 0.54, 95% CI: 0.33–0.88), gross motor (OR = 0.29, 95% CI: 0.12–0.71), problem solving (OR = 0.40, 95% CI: 0.19–0.81), and personal-social development (OR = 0.28, 95% CI. 0.17–0.47). Frequent egg intake was associated with decreased risk of delays in fine motor (OR = 0.44, 95% CI: 0.24–0.81) and personal-social (OR = 0.63, 95% CI: 0.40–0.98). A higher intake of livestock, fish and shrimp was significantly related to a reduced risk of delay in gross motor development (OR = 0.43, 95% CI: 0.20–0.93). Furthermore, moderate soy intake (1 time/week) was associated with a lower risk of delays in fine motor (OR = 0.47, 95% CI: 0.22–0.97), problem solving (OR = 0.19, 95% CI: 0.04–0.77), and personal-social development (OR = 0.45, 95% CI: 0.23–0.89). However, a higher intake of soy (≥2 times/week) was associated with an increased risk of delay in the problem-solving domain (OR = 2.07, 95% CI: 1.07–3.99).

## 4 Discussion

In this longitudinal cohort study, we examined the association between Infant and Child Feeding Index (ICFI) scores at 8 months and neurodevelopmental outcomes through age two. Higher ICFI scores, defined as exceeding 60% of the maximum, were significantly associated with reduced risk of developmental delay in the communication, problem-solving, and personal-social domains. Furthermore, when analyzed as a continuous variable, a significant relationship with reduced risk of delays in the communication, fine motor, and personal-social domains, reinforcing the robustness of these associations. The ICFI thus represents a promising composite indicator of infant feeding quality, with higher scores suggesting better adherence to recommended practices in this cohort. Conversely, the attenuation of the problem solving motor association after covariate adjustment suggests that this higher-order cognitive domain may be more susceptible to socioeconomic confounding. While meeting a feeding-quality threshold is beneficial, its incremental effect is less robust than the linear relationships seen with communication and motor skills. Our results corroborate those of Moursi et al. (26), who demonstrated that the ICFI effectively captures clustered positive or negative feeding behaviors and identifies threshold effects at which cumulative beneficial practices yield neurodevelopmental gains. A robust body of literature has linked early dietary patterns to later cognitive outcomes; for example, an Australian birth cohort found that healthy infant eating patterns, characterized by frequent consumption of whole grains, fruits, vegetables, quality proteins, and dairy, predicted superior neurocognitive performance at age 17 (27). However, few studies have examined the ICFI’s predictive value for neurodevelopment in children under 2 years of age. Our findings extend this evidence base by demonstrating that a comprehensive feeding index can identify infants at risk for early developmental delays, thereby supporting the ICFI’s utility in guiding targeted, nutrition-focused interventions during the critical first 2 years of life.

The ICFI encompasses several variables related to breastfeeding and complementary feeding practices. Our analysis of its components revealed that the absence of bottle feeding (reflecting direct feeding practices, which include direct breastfeeding) was significantly associated with a reduced risk of delays in communication and problem-solving skills. This finding aligns with a cohort study conducted in Singapore, which reported that infants who were directly fed by their mothers (at the breast) exhibited higher performance on several memory tasks compared to those who received breast milk via bottle-feeding, even after accounting for the nutrient composition of the milk. While our variable captured ‘bottle feeding’ in general (which may include formula or expressed breast milk), the observed protective effect of not using a bottle likely reflects, in large part, the benefits of direct feeding practices at the breast. One plausible mechanism is that direct breastfeeding facilitates more responsive feeding interactions, richer olfactory and tactile stimulation, and greater maternal-infant synchrony—all of which are thought to be instrumental in promoting neurodevelopment (28). Our results, therefore, indirectly support the WHO recommendation of direct breastfeeding whenever feasible (29), and suggest that the mode of milk feeding (bottle versus direct) is an important dimension of infant feeding practice. The consistency of protective associations when using the mean ICFI score from 8 and 12 month assessments further strengthens the evidence that sustained high-quality feeding practices, rather than isolated time-points, are important for optimal neurodevelopment.

Our study also revealed a nuanced relationship between the timing of formula supplementation and gross development in infants. Specifically, introducing formula between 4 and 6 months was associated with a reduced risk of delayed gross motor development compared to its introduction before 4 months. However, no significant association was observed when formula was added after 6 months of age. It is important to emphasize that this finding should not be interpreted as evidence that formula feeding is superior to breastfeeding. Rather, the observed association likely reflects the developmental risks of introducing formula too early. This is consistent with evidence that very early formula supplementation may disrupt the establishment of breastfeeding and deprive infants of the complete nutritional and bioactive components of breast milk, which are crucial for optimal neurological development (30–32). This interpretation aligns with our other finding that direct breastfeeding practices (i.e., absence of bottle feeding) were beneficial. Therefore, our results ultimately reinforce the recommendation of exclusive breastfeeding for the first 6 months, while providing nuanced evidence for timing considerations when formula is used. These insights highlight the complexity of infant feeding strategies and their long-term impacts on neurodevelopment. Future research should continue to explore the optimal timing and composition of complementary foods to best support the diverse nutritional needs of infants during this critical period of rapid growth and development.

Our findings indicate that children who exhibited higher dietary diversity and more frequent meals at 8 months of age demonstrated superior performance across several developmental domains. This observation aligns with our additional finding that increased consumption of fresh vegetables and fruits, eggs, livestock, fish and shrimp during the past week in infants was associated with a reduced risk of developmental delays in multiple domains. These results emphasize the importance of a varied and nutrient-rich diet in supporting early childhood development. Consistent with our observations, Miller et al. (33) conducted a series of six dietary surveys among preschool children in Nepal and found that higher dietary diversity scores were significantly associated with enhanced overall child development. Furthermore, a study conducted in China also found a negative dose-response relationship between the number of food groups consumed by infants and the risk of developmental delays up to 2 years of age (34). These studies highlight the dietary diversity has been an indicator of dietary quality in infants and young children.

The nutritional benefits of fruits and vegetables are well-established, providing a broad spectrum of essential micronutrients vital for growth and development. Notably, vegetables and fruits rich in vitamin A play a crucial role in maintaining adequate vitamin A levels, which are essential for vision, immune function, and cellular differentiation. Similarly, ascorbic acid (vitamin C)-rich vegetables and fruits act as a booster of non-heme iron and folic acid absorption (35, 36). These nutritional properties may underlie the observed association between early fruit and vegetable intake and subsequent cognitive development. Multiple longitudinal studies have established that greater consumption of vegetable and fruit during infancy predicts better cognitive performance later in childhood (13, 37, 38). Notably, Panossian et al. (39) reported that increased fruit intake at 1 year of age was prospectively associated with enhanced cognitive development at 10 years of age, suggesting long-term neurodevelopmental benefits of early dietary patterns.

Animal-source foods constitute a nutritionally dense component of infant diets, providing high-quality protein and essential micronutrients critical for growth and neurodevelopment. These foods are particularly rich in protein, iron, zinc, vitamin A, vitamin B12, and vitamin D. Notably, fish is rich in polyunsaturated fatty acids and iodine, both of which play crucial roles in neural development and cognitive function (39–42). Our findings align with longitudinal evidence demonstrating the developmental benefits of early animal-source food consumption. A cohort study (43) reported a significant positive association between meat intake and psychomotor development indices across critical neurodevelopmental windows (4–12 months and 4–16 months of age). These consistent observations underscore the importance of incorporating animal-source foods into complementary feeding regimens to support optimal neurocognitive development during infancy.

Beyond animal-derived foods, soy emerges as a significant plant-based protein source, offering potential cognitive benefits attributable to its high isoflavone content (44). Isoflavones, a class of phytoestrogens abundant in soy, have been implicated in neuronal protection against oxidative stress-a mechanism posited to underlie their favorable impact on neurodevelopment (45). In our study, soy consumption was relatively low at 25.1% among 8-month-old infants, and moderate intake was associated with reduced neurodevelopmental delays. Interestingly, we observed that higher soy consumption (≥2 days/week) was associated with an increased risk of delays in problem-solving skills. This unexpected finding may be attributed to the relatively low overall consumption of soy in our sample, potential measurement errors, or confounding by other dietary factors. Further research is needed to clarify the relationship between soy intake and specific neurodevelopmental domains.

Several limitations of this study should be acknowledged. First, the study may be subject to recall bias inherent in retrospective dietary assessments. To minimize this potential bias, all investigators received standardized training to ensure consistent administration of dietary surveys, and provided one-on-one assistance to participants to enhance recall accuracy. Second, variability of infant feeding practices represents another limitation. However, our sensitivity analysis demonstrated that the significant association between ICFI score and neurodevelopmental outcomes persisted when accounting for feeding practice at both 8 and 12 months of age (Supplementary Table 1), suggesting robustness of these findings. Third, the ICFI, as a composite index, has inherent methodological constraints. It is structured to reward the achievement of minimum dietary diversity and frequency but does not account for potential excessive intake of specific food groups (e.g., high consumption of red meat), which might have different health implications. This is a common limitation of such indices and suggests an area for future methodological refinement. Forth, despite adjustment for a range of known covariates, residual confounding from unmeasured variables, such as maternal nutritional status during pregnancy or lactation, home stimulation environment, supplement use and child care attendance may persist despite our adjustment for known covariates. Additionally, our dietary assessment did not specifically capture data on the consumption of processed foods. The role of food processing in early childhood neurodevelopment is an important and emerging area of research. Future prospective studies incorporating more comprehensive covariate assessments, as well as detailed evaluations of processed food intake, would provide a more holistic understanding of early diet quality and help confirm these associations. Finally, the generalizability of our findings should be considered. The Shenzhen Birth Cohort is comprised of an urban population with a relatively high socioeconomic status. While this provides valuable insights into this demographic, it may limit the direct application of our results to populations in rural settings or with significantly different cultural and socioeconomic backgrounds. Future studies should aim to validate these associations in more diverse populations. Despite these limitations, our study has several notable strengths, including its prospective longitudinal design, the use of a validated and comprehensive feeding index (ICFI), repeated neurodevelopmental assessments using the ASQ-3 across multiple time points, and the application of multiple analytical approaches to robustly examine the association between feeding practices and child development.

## 5 Conclusion

This study demonstrates a robust association between Infant and Child Feeding Index (ICFI) scores at 8 months and early childhood neurodevelopment. The ICFI scores serves as a valuable tool for assessing the feeding practice, which plays a pivotal role in fostering optimal neurodevelopment. Our findings indicate that specific feeding practices, including appropriate duration of exclusive breastfeeding, direct maternal breastfeeding, timely introduction of nutrient-rich complementary foods, and adequate feeding frequency, are associated with a reduced risk of neurodevelopmental delays across multiple domains during early childhood. These results underscore the importance of evidence-based nutritional guidance in early childhood to support healthy neurodevelopmental trajectories. Future research should focus on identifying critical windows of opportunity within the first 2 years of life when targeted interventions in feeding practices may yield the greatest benefits in mitigating the risk of neurodevelopmental delays. Such endeavors will contribute to the development of comprehensive strategies aimed at optimizing early childhood nutrition and enhancing long-term cognitive and developmental outcomes.

## Data Availability

The raw data supporting the conclusions of this article will be made available by the authors, without undue reservation.
