# Supplementary material for: Association between the Infant and Child Feeding Index at 8 months and early childhood neurodevelopment
Source: Front Nutr. 2026 Jan 14;12:1727649. doi: 10.3389/fnut.2025.1727649 (PMC12846993; doi:10.3389/fnut.2025.1727649)
Supplement: Supplementary file 1 [file Table_1.DOCX]

**Supplement Table.** Association between the mean ICFI scores at both 8 and 12 months of age and infants neurodevelopment (n = 649)

| ASQ | Mean ICFI values at 8 and 12 months of age(dichotomized) | | Mean ICFI values at 8 and 12 months of age(continuous) | |
| --- | --- | --- | --- | --- |
|  | OR (95% CI) | *P* | OR (95% CI) | *P* |
| **Communication** |  |  |  |  |
| Crude | **0.61 (0.41, 0.91)** | **0.017** | **0.93(0.88, 0.99)** | **0.013** |
| Adjusted | **0.64 (0.43, 0.97)** | **0.034** | **0.93(0.88, 0.99)** | **0.031** |
| **Gross motor** |  |  |  |  |
| Crude | 0.96 (0.53, 1.73) | 0.9 | **0.89(0.81, 0.99)** | **0.030** |
| Adjusted | 1.00 (0.55, 1.81) | >0.9 | **0.88(0.78, 0.98)** | **0.019** |
| **Fine motor** |  |  |  |  |
| Crude | **0.49 (0.30, 0.82)** | **0.007** | **0.91(0.85, 0.97)** | **0.002** |
| Adjusted | **0.47 (0.27, 0.81)** | **0.006** | **0.89(0.83, 0.95)** | **<0.001** |
| **Problem solving** |  |  |  |  |
| Crude | **0.51 (0.28, 0.92)** | **0.027** | **0.92(0.85, 0.98)** | **0.017** |
| Adjusted | **0.54 (0.30, 0.96)** | **0.039** | **0.91(0.84, 0.99)** | **0.033** |
| **Personal-social** |  |  |  |  |
| Crude | **0.59 (0.38, 0.91)** | **0.014** | **0.91(0.86, 0.96)** | **0.001** |
| Adjusted | **0.61 (0.40, 0.93)** | **0.02** | **0.91(0.85, 0.97)** | **0.002** |

| The mean ICFI scores <13.8 was used as the control group. Adjusted for: monthly household income; maternal age, education attainment, ethnicity, household registration, parity; delivery mode; child’s sex, and small-for-gestational-age (SGA) status. |
| --- |
